# Multiorgan involvement with cardiac, renal and skeletal manifestations following inhalation of compressed air: A case report

**DOI:** 10.3892/mi.2026.298

**Published:** 2026-01-20

**Authors:** Parth Adrejiya, Sana Irshad, Mohammad Abubaker, Negarsadat Neshat, Srikanth Maddika, Muthusamy Sekar

**Affiliations:** 1 Department of Internal Medicine, Wellstar Spalding Regional Hospital, Griffin, GA 30224, USA; 2 Department of Internal Medicine, Wellstar Kennestone Hospital, Marietta, GA 30060, USA; 3 Department of Internal Medicine (Faculty), Wellstar Spalding Regional Hospital, Griffin, GA 30224, USA; 4 Department of Interventional Cardiology (Faculty), Wellstar Spalding Regional Hospital, Griffin, GA 30224, USA

**Keywords:** inhalant abuse, hydrofluorocarbon toxicity, troponin elevation, multiorgan dysfunction, compressed air duster cans

## Abstract

The abuse of inhalants, such as compressed air duster cans containing hydrofluorocarbons (i.e., tetrafluoroethane and difluoroethane) is an underrecognized cause of multisystem organ dysfunction. The present study describes the case of a 29-year-old male patient presenting with syncope, elevated levels of troponins, acute kidney injury and diffuse bony sclerosis following acute on chronic daily inhalation of compressed air. A cardiac evaluation revealed troponin elevation attributed to non-thrombotic myocardial injury (type 2 MI) related to hydrofluorocarbon toxicity, rather than a classic type 1 acute coronary syndrome, while imaging and laboratory analyses suggested skeletal involvement with possible toxic bone disease. The patient was treated conservatively with supportive measures, with successful resolution of his laboratory abnormalities as well as other symptoms. To the best of our knowledge, the case described herein represents one of the few reported cases in the literature linking hydrofluorocarbon inhalant abuse to such widespread systemic complications. The present case report highlights the broad spectrum of organ injury linked to compressed air duster cans and underscores the importance of considering dust inhalant toxicity in young adults presenting with unexplained cardiac biomarkers and systemic abnormalities.

## Introduction

The National Institute on Drug Abuse (NIDA) reports that inhalants continue to be one of the most commonly abused substances in the USA (1). They are used through methods such as sniffing, huffing, or bagging and often involve easily accessible products such as compressed air duster cans containing hydrofluorocarbons, including tetrafluoroethane and difluoroethane. These chemicals induce rapid psychoactive effects via gamma-aminobutyric acid enhancement and N-methyl-D-aspartate receptor receptor suppression; however, their overall toxic effects are not yet fully defined (2). Compressed air duster cans are widely available inhalants composed of liquefied hydrofluorocarbon propellants, most commonly difluoroethane. These products are intended for cleaning electronic equipment, but can produce significant toxicity when misused. Safety data for these agents warn that concentrated inhalation may lead to respiratory irritation, altered mental status, cardiac conduction abnormalities, arrhythmias, impaired circulation, loss of consciousness and even sudden death. High-level exposure has also been shown to be associated with renal dysfunction and other systemic complications (3,4). Although the majority of reported cases involve individuals aged between 18 and 50 years, the 2019 National Youth Risk Behavior Survey noted that ~6.5% of younger students had engaged in the use of inhalants, highlighting early-age vulnerability (5).

The present study describe the case of a young male patient with severe multi-organ toxicity (cardiac, renal and skeletal) following chronic compressed air duster use, who exhibited significant improvement with supportive care and was ultimately discharged. The present case report underscores the need for clinicians to recognize the varied presentations of duster abuse to ensure timely diagnosis and appropriate acute management.

## Case report

A 29-year-old Caucasian male with a history of chronic alcohol use disorder, daily tetrahydrocannabinol use, asthma, hypertension and psychiatric comorbidities presented to the Emergency Department of Wellstar Spalding Regional Hospital (Griffin, USA) May 12, 2025 following a syncopal episode. He described several days of progressive weakness, dizziness and joint pain requiring cane-assisted ambulation. On the day of admission, he collapsed while descending stairs. This event was witnessed, and there was no evidence of seizure activity or post-ictal confusion. He admitted to the extensive daily use of compressed air duster cans for several months, experiencing euphoria and increasing nausea with each use. On the day of presentation, he had used 8 cans of dust inhalant, instead of 4 as per his routine use.

**Figure 1:**
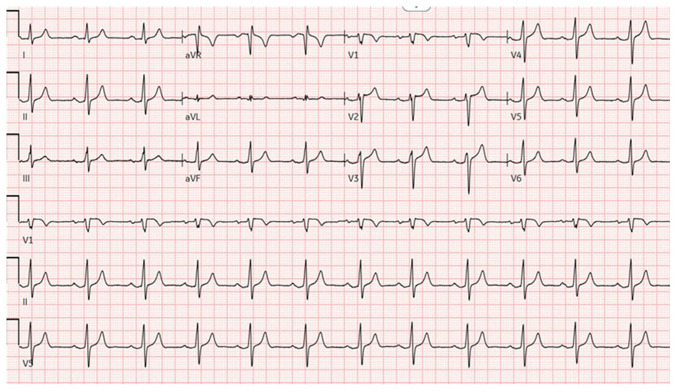
12-lead electrocardiogram illustrating normal sinus rhythm with peaked T waves in leads in V2, V3, V4 and V5, and PR interval prolonged 206 msec.

**Figure 2:**
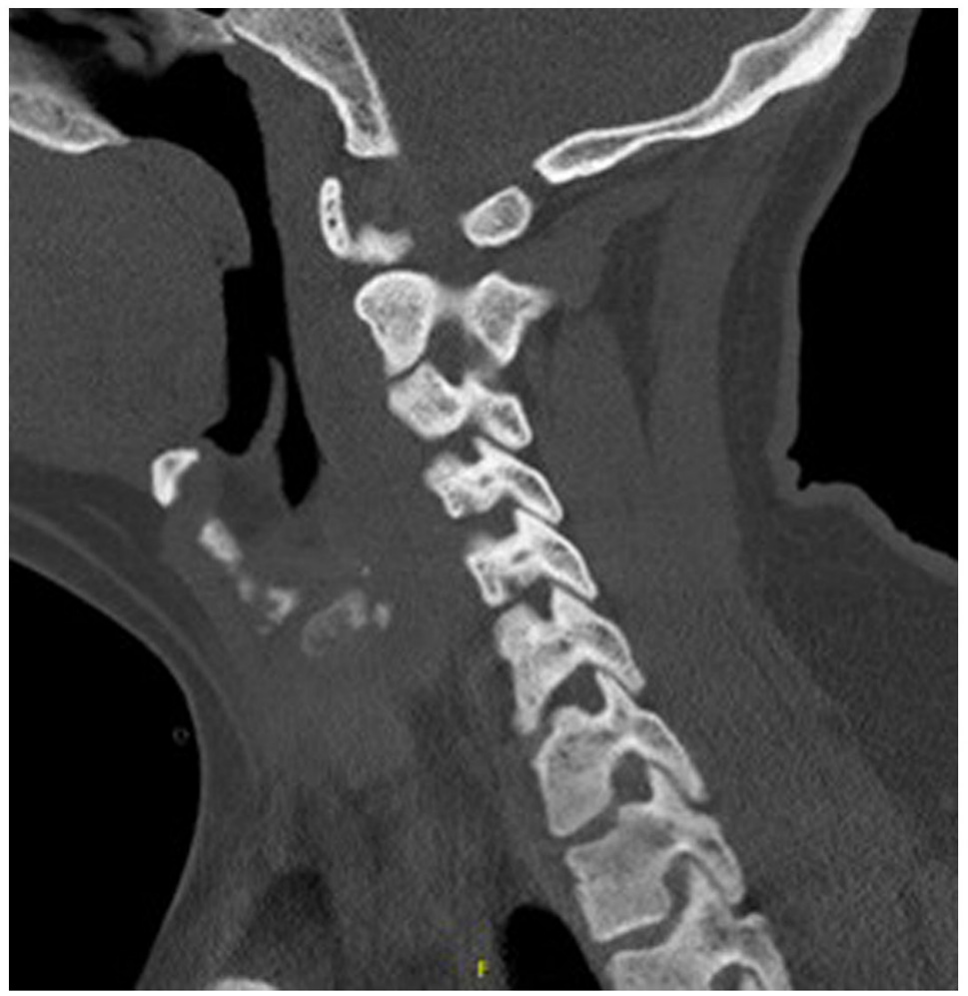
Computed tomography scan of the cervical spine without IV contrast illustrating generalized osteosclerosis.

**Figure 3:**
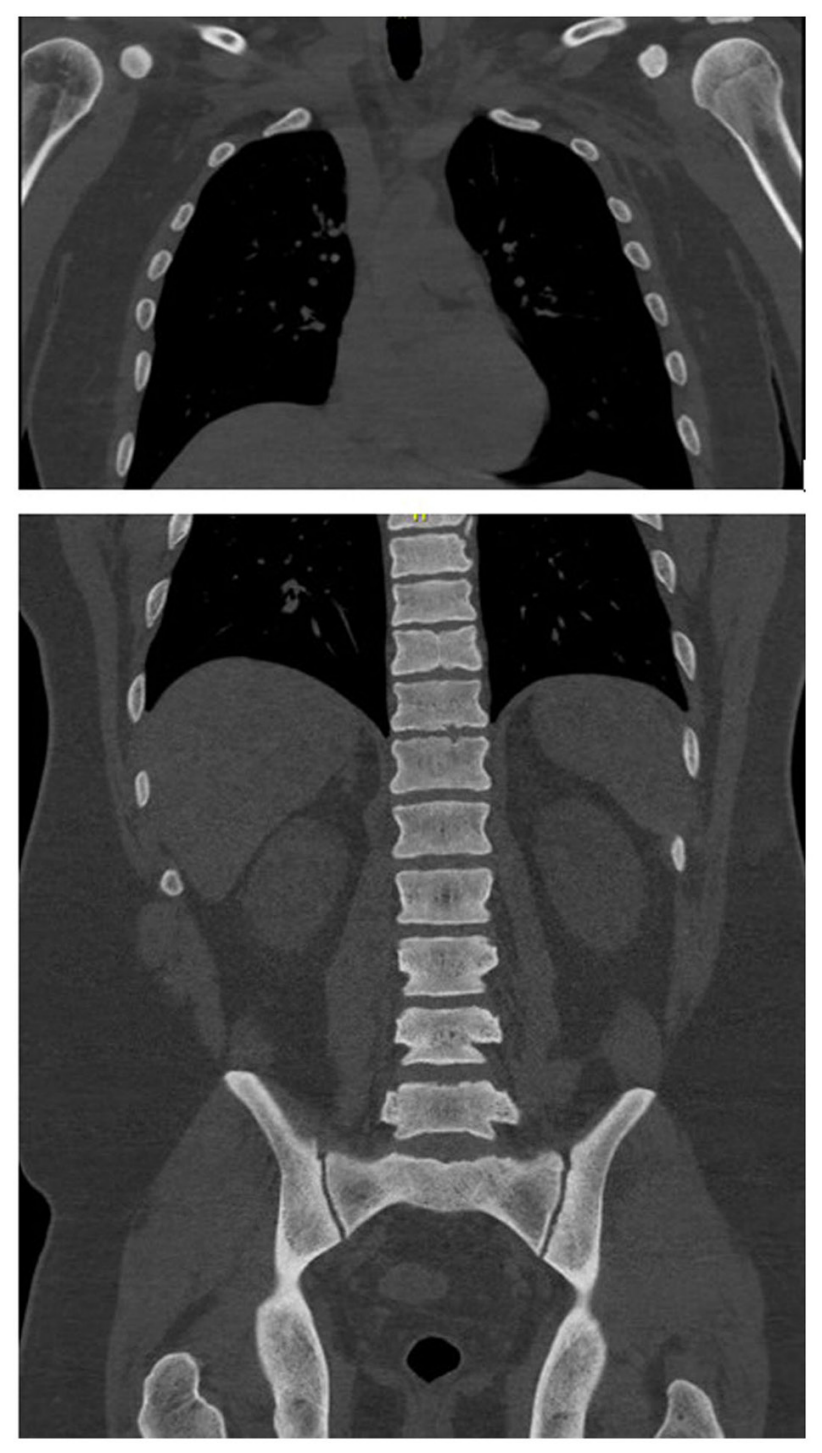
Computed tomography scan of the chest (upper panel), and abdomen and pelvis (lower panel) without IV contrast bone window illustrating the diffuse sclerosis of bony structures in the humerus head, thoracolumbar vertebrae and pelvic bones.

Upon arrival, he was hypotensive and symptomatic. An electrocardiogram (Fig. 1) demonstrated sinus rhythm with peaked T waves. Laboratory results revealed hyperkalemia, elevated levels of troponins (in thousands; ng/l), acute kidney injury and high anion gap metabolic acidosis. Urine drug screening yielded negative results, while salicylate and acetaminophen levels were normal. Other laboratory results are listed in Table I. Imaging analyses, including a computed tomography scan of the cervical spine (Fig. 2), and chest, abdomen and pelvis (Fig. 3), revealed diffuse bony sclerosis.

He was managed with intravenous fluids, a heparin drip 10 units/kg/h for 24 h; aspirin at 325 mg, one dose; atorvastatin at 80 mg for 2 days; a hyperkalemia protocol including 10 ml of 10% calcium gluconate, 1 dose and 10 mg nebulized albuterol, one dose; and sodium bicarbonate infusion at 100 ml/h for 1 day. Cardiology attributed the troponin elevation to non-thrombotic myocardial injury (type 2 MI) rather than acute plaque rupture, given the clinical context and normal ventricular function on an echocardiogram. Therefore, the heparin drip was discontinued after 24 h. The patient was monitored on telemetry and had no arrhythmias.

Nephrology and Hematology-Oncology were consulted to investigate potential underlying etiologies for his acute kidney injury and diffuse skeletal sclerosis. A comprehensive differential diagnosis was considered and methodically ruled out. Multiple myeloma was excluded with negative serum and urine protein-to-creatinine ratio (UPCR) and immunofixation analyses. Renal osteodystrophy was deemed unlikely due to preserved renal function on follow-up and lack of secondary hyperparathyroidism. Glomerulonephritis and vasculitis were excluded via negative serologic and urinary studies. No evidence of paraneoplastic syndrome or infiltrative bone marrow disorder was found, and vitamin D deficiency and prostate-specific antigen levels were within normal range. Imaging and laboratory trends supported a toxic-metabolic etiology, most likely secondary to chronic inhalant exposure. The patient improved with supportive therapy, with laboratory values returning to levels within normal limits; he was discharged after 3 days with referrals to nephrology, hematology-oncology and addiction medicine.

## Discussion

1,1-Difluoroethane, a hydrofluorocarbon propellant found in a number of compressed-gas cleaning products, is frequently misused for its rapid onset of central nervous system depression and transient euphoria. Owing to its high lipid solubility, the compound readily crosses the blood-brain barrier, producing effects that last only a few minutes. Its easy availability, low cost and short-lived intoxication render difluoroethane-containing compressed air duster cans particularly attractive for recurrent recreational use (6). The patient described herein reported inhaling approximately four cans daily for several months and doubled this amount to eight cans on the day of presentation, highlighting both tolerance and escalating exposure patterns typical of chronic inhalant misuse.

Reported neurologic manifestations of difluoroethane and other inhalant exposure range from mild to severe and may include drowsiness, headaches, gait instability, dizziness, visual disturbances, generalized weakness, profound fatigue, lethargy, altered consciousness, seizures and even coma. Hypoxia resulting from oxygen displacement within the alveoli or aspiration-related lung injury can further exacerbate central nervous system depression. Chronic users may also experience lasting effects, such as cognitive deficits, cerebellar dysfunction and peripheral neuropathy (7). In the case described herein, the inhalant use of the patient culminated in a syncopal episode, consistent with the acute neurologic and systemic instability described in the literature (4).

Marked cardiovascular complications from hydrocarbon inhalation include myocardial dysfunction and life-threatening arrhythmias. Halogenated hydrocarbons, in particular, have been shown to be associated with fatal ventricular dysrhythmias and the phenomenon of ‘sudden sniffing death’, an unpredictable event reported even in first-time users. This is deemed to result from increased myocardial sensitivity to catecholamines, compounded by inhalant-induced hypoxia, which promotes delayed after-depolarizations and malignant ventricular arrhythmias (8-12). In the patient in the present study, inhalant use was accompanied by severe hyperkalemia with corresponding electrocardiogram changes, syncope and markedly elevated troponin levels in the thousands, although echocardiography demonstrated preserved systolic function without structural abnormalities.

Cao *et al* (3) reported the case of a 35-year-old chronic duster user who developed ischemic-appearing electrocardiogram changes and elevated troponin, ultimately found to have non-obstructive coronaries on catheterization, consistent with inhalant-related cardiac injury. Similarly, the patient in the present study exhibited electrocardiogram abnormalities most notably peaked T waves along with syncope and significant troponin elevation. Similarly, in the patient described herein, the increase in troponin levels was ultimately deemed type 2 MI (supply-demand mismatch) or toxic myocardial injury, rather than a classic type 1 infarction. As the clinical suspicion for true coronary disease was low and echocardiography was normal, cardiac catheterization was deferred. The Fourth Universal Definition of myocardial infarction (13) emphasizes that troponin elevation without evidence of acute atherothrombosis should be classified as myocardial injury or type 2 MI (due to this, his heparin was terminated and angiography was deferred). Hydrocarbon toxicity can also cause myocarditis; cardiac MRI in such cases presents as inflammation; however, in the patient in the present study, the echo was normal and he had no focal wall motion abnormalities. In summary, the clinical picture favors a toxic myocardium from inhalants (type 2 MI) over an acute plaque rupture infarct (13). Long *et al* (14) previously described one of the earliest reported cases of acute renal failure associated with duster inhalation. Similar to that report, the patient described herein also presented with acute kidney injury, which improved significantly with intravenous fluid resuscitation. Although dehydration likely contributed to his renal dysfunction, a direct nephrotoxic effect of difluoroethane cannot be fully excluded.

Inhalant-related skeletal fluorosis can produce diffuse osteosclerosis, trabecular coarsening, cortical thickening and soft-tissue ossifications, with imaging serving as the most sensitive diagnostic tool. As fluoride accumulates in bone with a long half-life, radiographic changes may persist for years even after exposure stops (15). In the patient in the present study, extensive osteosclerosis was evident despite his young age, consistent with chronic hydrofluorocarbon exposure, although a DEXA scan was not obtained. However, in the present study, serum or urine fluoride levels were not measured to confirm fluorosis; thus, this is a limitation of the present study. Without fluoride quantification, a definitive diagnosis of skeletal fluorosis cannot be made. Instead, toxic-metabolic bone disease from inhalant exposure was considered more likely. The differential diagnosis for diffuse osteosclerosis is limited, but includes osteoblastic metastases (e.g., prostate and breast), sclerotic myeloma, myelofibrosis, mastocytosis, Paget's disease and rare granulomatous disorders, such as sarcoidosis. In the case described herein, malignancy and myeloma were investigated and excluded, and there was no evidence of systemic diseases (such as sarcoidosis) that may cause similar bone changes.

In conclusion, the present study describes the case of a 29-year-old male patient who developed syncope after inhaling multiple compressed air duster cans, presenting with acute cardiac injury, renal dysfunction and diffuse skeletal sclerosis. His findings parallel prior reports of multisystem toxicity from hydrofluorocarbon inhalation (3,5,6,14,15). Although the current evaluation was limited by the absence of cardiac catheterization and DEXA imaging, the clinical picture strongly supports inhalant-induced organ injury. Clinicians should consider duster abuse in young patients with unexplained syncope, electrocardiogram abnormalities, or multiorgan dysfunction. Ongoing research is required in order to better define the pathophysiology and long-term effects of these increasingly common exposures.

## Figures and Tables

**Table I:** Laboratory values of the patient upon admission.

Laboratory test	Patient value	SI unit	Reference range
Sodium (Na)	129.0	mmol/l	135-145 mmol/l
Chloride (Cl)	102.0	mmol/l	98-106 mmol/l
Potassium (K)	7.3	mmol/l	3.5-5.1 mmol/l
Bicarbonate (HCO_3_)	14.0	mmol/l	22-28 mmol/l
Phosphorus (PO_4_)	1.1	mg/dl	2.5-4.5 mg/dl
Calcium	10.1	mg/dl	8.4-10.2 mg/dl
BUN	45.0	mg/dl	7-20 mg/dl
Creatinine	2.49	mg/dl	0.6-1.3 mg/dl
Anion gap	20.0	mmol/l	8-16 mmol/l
Creatine kinase (CK)	361.0	U/l	38-174 U/l
Troponin	1062.0	ng/l	<14 ng/l
Pro-BNP	416.0	pg/ml	<125 pg/ml
Alkaline phosphatase (ALP	292.0	U/l	44-147 U/l
ALP bone fraction	96.0	U/l	28-66 U/l
ALP intestinal fraction	0.0	U/l	1-24 U/l
ALP liver fraction	14.0	U/l	25-69 U/l
Haptoglobin	770.0	mg/dl	30-200 mg/dl
Lactic acid	1.0	mmol/l	0.5-2.2 mmol/l
Vitamin D	39.0	ng/ml	30-100 ng/ml
Hemoglobin	12	g/dl	13.5-17.5 g/dl
Ferritin	1255.0	ng/ml	20-500 ng/ml
Iron	66.0	µg/dl	60-170 µg/dl
MCV	95.0	fl	80-100 fl
Folate	7.3	ng/ml	3.1-17.5 ng/ml
Vitamin B12	655.0	pg/ml	200-900 pg/ml
Platelets	550.0	x10^3^/µl	150-400x0^3^/µl
ESR	90.0	mm/h	0-20 mm/h
INR	1.38		0.8-1.2
TSH	1.01	µIU/ml	0.4-4.0 µIU/ml
Cardio CRP	8.4	mg/l	<3.0 mg/l
Complement C3	200.0	mg/dl	90-180
Complement C4	37.0	mg/dl	10-40
PSA	0.2	ng/ml	0-4.0
PTH	25	pg/ml	10-65
ANA	Negative		Negative
ANCA	Negative		Negative
Hepatitis panel	Negative		Negative
HIV	Negative		Negative
UPCR	1,269 High		22-128 mg/g
UA microscopy	1+ blood, 2+ protein		None
SPEP/UPEP with immunofixation	No paraprotein detected		No paraprotein detected
Serum free light chains	Not detected		Not detected
Urine drug screen	Not detected		Not detected
Serum salicylate/acetaminophen	Not detected		Not detected

BUN, blood urea nitrogen; Pro-BNP, pro-B-type natriuretic peptide; MCV, mean corpuscular volume; ESR, erythrocyte sedimentation rate; INR, international normalized ratio; TSH, thyroid stimulating hormone; CRP, C-reactive protein; PSA, prostate-specific antigen; PTH, parathyroid hormone; ANA, antinuclear antibodies; ANCA, antineutrophil cytoplasmic antibody; HIV, human immunodeficiency virus; UPCR, urine protein-to-creatinine ratio; SPEP, serum protein electrophoresis; UPEP, urine protein electrophoresis.

## Data Availability

The data generated in the present study may be requested from the corresponding author.
